# A Comparative Analysis of Explainable Artificial Intelligence Models for Electric Field Strength Prediction over Eight European Cities

**DOI:** 10.3390/s25010053

**Published:** 2024-12-25

**Authors:** Yiannis Kiouvrekis, Ioannis Givisis, Theodor Panagiotakopoulos, Ioannis Tsilikas, Agapi Ploussi, Ellas Spyratou, Efstathios P. Efstathopoulos

**Affiliations:** 1 Mathematics, Computer Science and Artificial Intelligence Lab, Faculty of Public and One Health, University of Thessaly, 43100 Karditsa, Greece; jgivisis@gmail.com; 2 Department of Information Technologies, University of Limassol, 3020 Limassol, Cyprus; 3 Business School, University of Nicosia, 46 Makedonitissas Avenue, 2417 Nicosia, Cyprus; 4 Department of Management Science and Technology, University of Patras, 26334 Patras, Greece; tpanagiotakop@upatras.gr; 5 Department Applied Physics and Mathematics, National Technical University of Athens, Iroon Polytechniou 9, Zografou, 15772 Athens, Greece; tsilikasgiannis@hotmail.com; 6 2nd Department of Radiology, Medical School, National and Kapodistrian University of Athens, 12462 Athens, Greece; aplousi@gmail.com (A.P.); spyratouellas@gmail.com (E.S.); stathise@med.uoa.gr (E.P.E.)

**Keywords:** machine learning, explainable artificial intelligence, electric field strength, radio environment map, urban areas, IoT sensors

## Abstract

The widespread propagation of wireless communication devices, from smartphones and tablets to Internet of Things (IoT) systems, has become an integral part of modern life. However, the expansion of wireless technology has also raised public concern about the potential health risks associated with prolonged exposure to electromagnetic fields. Our objective is to determine the optimal machine learning model for constructing electric field strength maps across urban areas, enhancing the field of environmental monitoring with the aid of sensor-based data collection. Our machine learning models consist of a novel and comprehensive dataset collected from a network of strategically placed sensors, capturing not only electromagnetic field readings but also additional urban features, including population density, levels of urbanization, and specific building characteristics. This sensor-driven approach, coupled with explainable AI, enables us to identify key factors influencing electromagnetic exposure more accurately. The integration of IoT sensor data with machine learning opens the potential for creating highly detailed and dynamic electromagnetic pollution maps. These maps are not merely static snapshots; they offer researchers the ability to track trends over time, assess the effectiveness of mitigation efforts, and gain a deeper understanding of electromagnetic field distribution in urban environments. Through the extensive dataset, our models can yield highly accurate and dynamic electric field strength maps. For this study, we performed a comprehensive analysis involving 566 machine learning models across eight French cities: Lyon, Saint-Étienne, Clermont-Ferrand, Dijon, Nantes, Rouen, Lille, and Paris. The analysis incorporated six core approaches: k-Nearest Neighbors, XGBoost, Random Forest, Neural Networks, Decision Trees, and Linear Regression. The findings underscore the superior predictive capabilities of ensemble methods such as Random Forests and XGBoost, which outperform individual models. Simpler approaches like Decision Trees and k-NN offer effective yet slightly less precise alternatives. Neural Networks, despite their complexity, highlight the potential for further refinement in this application. In addition, our results show that the machine learning models significantly outperform the linear regression baseline, demonstrating the added value of more complex techniques in this domain. Our SHAP analysis reveals that the feature importance rankings in tree-based machine learning models differ significantly from those in k-NN, neural network, and linear regression models.

## 1. Introduction

### 1.1. The Motivation

The motivation for this research is grounded in two distinct fields: the first is the field of health and the second is the field of telecommunications. The strength of the electric field and its potential health effects, particularly in relation to radiofrequency (RF) and non-ionizing radiation, remain the subject of ongoing research [1,2]. Although electric fields can generate low level currents within the human body, which are generally considered harmless, higher RF and microwave exposure levels can result in tissue heating [3,4]. The potential link between long-term high-level exposure to RF and cancer, especially brain tumors, continues to be the focus of active research [5]. The International Agency for Research on Cancer (IARC) has classified RF electromagnetic fields as “possibly carcinogenic to humans” (Group 2B). Additionally, some research suggests a possible association between electromagnetic field (EMF) exposure and neurological issues such as headaches, sleep disturbances, and cognitive impairment, although the evidence remains inconclusive [6]. Some individuals report symptoms such as headaches, fatigue, and stress, which they attribute to exposure to EMF. Although the World Health Organization (WHO) does not recognize electromagnetic hypersensitivity (EHS) as a medical diagnosis, the reported symptoms are real and can be severe for those affected. Regulatory organizations, including the International Commission on Non-Ionizing Radiation Protection (ICNIRP) and the Federal Communications Commission (FCC), have established safety guidelines for exposure based on scientific evidence, and these guidelines are periodically reviewed to incorporate new research findings [7]. The expansion of 5G technology has also raised public concerns about its potential health impacts [8,9]. Consequently, public health agencies continue to emphasize the need for ongoing research on the long-term health effects of RF exposure and advocate for evidence-based guidelines to ensure public safety.

Radio Environment Maps (REMs) are digital tools that provide detailed visualizations of radio frequency environments within specific geographic areas. These maps facilitate the analysis and optimization of radio signal propagation and interaction with the surrounding landscape [10,11]. The construction of REMs is a complex process that involves the collection of essential information, where the processing and preparation of newly derived information for an accurate outcome remain an open challenge. The REM construction process uses both signal measurements and methods to estimate signal levels in geographic locations where measurements are not available. Therefore, the highlighted challenge lies in the development of reliable and efficient methods for creating Radio Environment Maps (REMs), a complex process that requires the collection, processing, and precise estimation of information [12]. Specifically, the construction of an accurate REM faces difficulties due to the need to estimate signal levels in areas with limited or missing measurements. The challenge is to find appropriate methods (direct, indirect, or hybrid) that can effectively utilize the data, ensuring high resolution and quality in the final map output [13]. Furthermore, the quality of an REM is highly dependent on the precision and consistency of the input data, which makes it essential to develop methods that can perform reliably even when working with data of variable quality or origin [14].

### 1.2. Objectives

Our objectives cover several key areas. First, we aim to foster a deeper understanding of the relationship between electric field strength and human health through continuous research and monitoring. Second, we seek to implement robust monitoring IoT systems to ensure that exposure limits are not exceeded, thus mitigating potential health risks. By promoting informed regulations and public awareness, we can work toward a future where the public is protected from the adverse effects of electromagnetic radiation.

Many urban areas lack detailed maps of the strength of the electric field, which impedes informed decision-making processes. This research aims to address this critical gap by introducing a pioneering machine learning (ML) approach. Our methodology extends beyond conventional prediction or estimation of electromagnetic field strength. Using explainable artificial intelligence (XAI) techniques, we provide valuable insight into the underlying factors that influence field strength. To validate our model, we used datasets from five diverse cities.

The cornerstone of our research lies in our comprehensive dataset, which includes not only field strength measurements but also critical factors such as population density, urbanization levels, and detailed building information. We train a diverse range of machine learning (ML) models, including k-nearest neighbors, neural networks, and decision trees, on this rich dataset to generate highly accurate electric field strength maps.

However, the value of our work extends beyond just map generation. Using explainable AI (XAI) techniques, we delve deeper into the underlying factors influencing electromagnetic exposure within urban environments. This transparency empowers city planners, policymakers, and researchers to gain a nuanced understanding of the relationship between urban infrastructure and electromagnetic field distribution, ultimately enabling them to make informed decisions for the creation of safer and more sustainable cities.

Our research on predicting electric field strength in urban areas is groundbreaking for the following reasons:

- By utilizing explainable artificial intelligence, we address the challenge that increasing the resolution of any dimension improves result accuracy and explainability. These models transcend the simple estimation of the strength of the electric field, uncovering the pivotal urban factors—population density, building characteristics, and levels of urbanization—that exert a substantial impact on the strength of the electric field.
- Our rich dataset addresses the need for carefully designed samples of large-dimensional sets consisting of frequency, spatial, and geographic information.
- Our research goes a step further by generating detailed electric field strength maps for large urban areas. These maps are not just informative; they are actionable tools. They empower individuals, organizations, and policy makers to make informed decisions about urban planning, public health initiatives, and proactive risk management strategies.
- Using the results of explainable AI and understanding these key determinants, policy makers are empowered to develop targeted and effective interventions. This can involve creating regulations or urban design strategies that minimize the potential risks associated with RF radiation.

## 2. Related Work

Levie et al. [15] proposed an efficient deep learning method for estimating the propagation path loss from a point *x* to any point *y* on a planar domain and they additionally proved that properly designed deep neural networks can learn how to estimate the path loss function, given an urban environment, in a very accurate and computationally efficient manner. In another study [16], the authors proposed a radio propagation prediction model using deep learning convolutional neural networks (CNNs). Chaves-Vilota et al. [17] presented a new machine learning framework called the DeepREM framework that comprises two deep learning models, U-net and conditional GAN (CGAN), designed to estimate radio environment maps (REMs) using sparse measurement data without requiring additional input data during operation. In addition, another team of investigators [18] introduced LocUNet, a convolutional neural network (CNN) specifically designed for the localization task, trained from end to end, and capable of accurately estimating a user’s position based on signal strength (RSS) received from a limited number of base stations (BSs). LocUNet leverages estimated path loss radio maps of the BSs along with RSS measurements from the users to determine their locations. This approach allows LocUNet to achieve state-of-the-art accuracy while maintaining high robustness against potential inaccuracies in radio map estimations, which is particularly beneficial in urban scenarios where signal conditions can be unpredictable. Wen et al. [19] explored a multisource domain adaptive GNN for regression (GNN-MDAR) to predict and complete the target radio environment map (REM) using multiple complete REMs and sparse spectrum monitoring data from the target domain. Molina-Tenario et al. [20] aimed to determine the number of primary users in cognitive radio networks, their carrier frequency, bandwidth, and the spectral gaps in the sensed spectrum of a specific area through the construction of radioelectric environment maps (REMs). The authors compared the results of classical digital signal processing methods and neural networks performed by the central entity. The results showed that the best-performing cognitive radio network was the one using neural networks, as they accurately detected primary users in terms of carrier frequency and bandwidth. In one study [21], the authors combined the Siamese neural network and an attention mechanism in computer vision, proposing an update mechanism based on the amount of wireless environmental change starting from image data to increase REM accuracy, especially when the wireless environment changes significantly and the REM is not updated promptly. A novel algorithm [22] was also presented for propagation attenuation maps based on a parabolic equation method, which allowed the demodulation of signal attenuation along the expected propagation direction. Finally, Chen et al. [23] applied a graph neural network (GNN) to construct an accurate radio map in an urban environment by transforming spatially sparse measurement points into a graph structure and extracting connectivity relationships using the building map. As shown in the related work Table 1, our work is innovative and unique as it is the only one that examines the comparison of machine learning models over such a large area. Moreover, this area includes eight cities, collectively exceeding 50 km^2^, considering that Paris alone, without its suburbs, exceeds 30 km^2^.

## 3. Materials and Methods

### 3.1. Dataset

**Figure 1:**
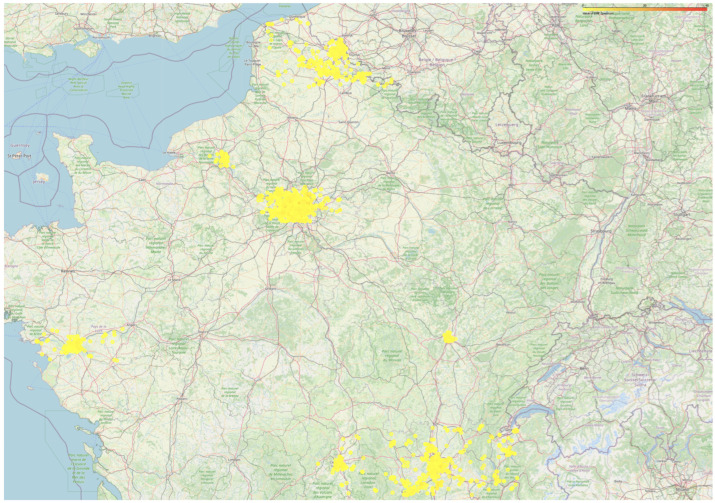
The distribution of the measurement points over France.

**Figure 2:**
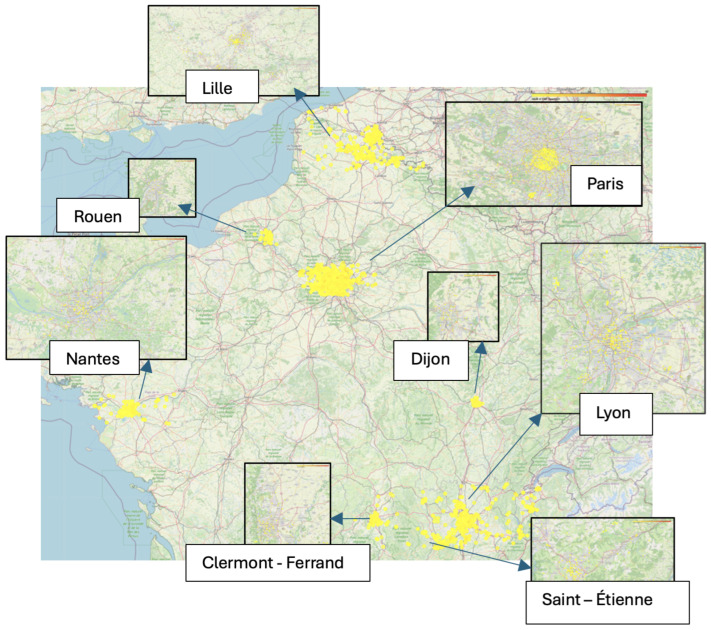
The distribution of the measurement points over the cities.

Our research benefits from a comprehensive dataset provided by the Agence nationale des fréquences (ANFR), a French governmental organization tasked with managing and regulating radio frequencies across the nation. Specifically, we used a dataset comprising 6547 electric field strength measurements collected from 2022 through the first semester of 2024 in eight major cities in France: Lyon, Saint-Étienne, Clermont-Ferrand, Dijon, Nantes, Rouen, Lille, and Paris (illustrated in Figure 1 and Figure 2).

This dataset was collected in strict adherence to Article L.34-9-1 of the French Post and Electronic Communications Code, which establishes a robust framework for data acquisition. Measurements were carried out for frequencies between 100 KHz and 6 GHZ, including mobile telecommunication systems at various frequencies, FM, radio broadcasting, television (TV), professional radio networks (PMRs), HF services (short-wave, medium-wave, and long-wave), radar, Wi-Fi, and cordless phones (DECT). A spatial average value of electric field strength (V/m) was measured over a six-minute time interval. At least three measurement points were used with respect to the average of a human body: at $h_{1}=1.70$ m, $h_{2}=1.50$ m, and $h_{3}=1.10$ m. Their analysis shows a median global exposure level of 0.38 V/m. The vast majority of these exposure levels were below 1 V/m. The measured levels were all well below the regulatory limits, which range between 28 V/m and 87 V/m depending on the frequencies. Compliance with the exposure limits for electromagnetic fields in the 100 kHz–6 GHz range, as defined by decree no. 2002-775 on 3 May 2002 was confirmed for all sites where measurements were taken. These guidelines ensure that all measurements are performed with precision, consistency, and compliance with the latest best practices in scientific, technical, and standardization. Consequently, the dataset offers a high level of reliability, making it a valuable resource for advancing research on electromagnetic field exposure and its implications. To prepare the dataset for analysis, we utilized a suite of robust Python libraries, which offer flexibility and advanced tools for data manipulation and visualization. These libraries allowed us to effectively organize and present the results. The finalized dataset includes eight critical variables, each of which provides valuable insight into the study.

**ID:** A unique identifier assigned to each antenna location, ensuring the traceability and organization of the dataset.
**EMF (V/m):** Represents the total electromagnetic field strength measured at each antenna, aggregated in all frequency bands.
**Urbanization Degree:** A categorical variable coded with eight levels, based on the classification system described in [24], indicating the degree of urbanization in the area surrounding the antenna.
**Population (people per 100 × 100 m):** Extracted from a detailed population density dataset [25], this variable provides the number of individuals residing within a 100 × 100 m square around each antenna.
**Built-up Volume (m^3^):** Derived from a spatial dataset [26], this variable quantifies the total volume of buildings in the vicinity of the antenna, reflecting the density of the built environment.
**Built-up Surface (m^2^):** This variable, obtained from another spatial dataset [27], measures the total surface area of buildings, encompassing residential, commercial, and other structures in the area.
**Building Height (m):** Based on a dataset focused on building heights [28], this variable indicates the average height of structures near each antenna, providing insight into the vertical development of the area.
**Settlement Characteristics:** Sourced from the GHS-BUILT-C dataset [29], this variable offers a comprehensive description of the inner structure and functionality of the built environment surrounding each antenna, capturing nuances of spatial planning and land use.

This structured dataset provides a multidimensional view of the environment surrounding each antenna, forming a solid foundation for in-depth analysis and interpretation of electromagnetic field measurements.

### 3.2. Preprocessing Data

**Figure 3:**
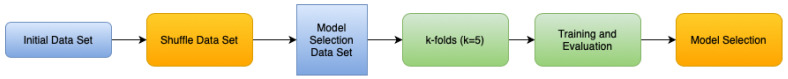
The methodology’s flowchart.

The diagram (Figure 3) represents a systematic workflow for data processing and model selection in a machine learning pipeline. It begins with the initial dataset, where the raw data are collected and prepared for analysis. The first step involves shuffling the dataset to randomize the order of the data, ensuring that the inherent patterns in the dataset order do not bias the training or evaluation process. After shuffling, the model selection dataset is created by preparing the data for further use, typically through splitting into subsets for training and validation. The workflow then uses a k-fold cross value (k = 5), where the dataset is divided into 50 subsets. In each iteration, the model is trained on four folds and evaluated on the remaining fold, with the process repeating five times to ensure a robust assessment of the model’s performance. During the training and evaluation phase, machine learning models are trained on the training data and assessed based on specific performance metrics. Finally, the process culminates in model selection, where the best-performing model is chosen based on its evaluation results, ensuring that the model with the highest reliability and accuracy is selected for deployment. This workflow ensures a thorough and unbiased approach to model development and selection. Before entering model training, we take steps to ensure that our data are clean and ready to use (Figure 3). First, we shuffle the initial dataset to prevent feature clustering and ensure representative sampling of all possible conditions. For the model selection process, we utilize a combination of two methodologies to evaluate our machine learning algorithms: the shuffling method and 5-fold cross-validation. Initially, we randomize the order of entries within the model selection dataset, then split the data into training and validation pairs using an 80–20% split ratio. Next, we divide the training dataset into five subsets, applying the 5-fold cross-validation method. Standardization, as described in Figure 3, is performed for each split during the 5-fold cross-validation. This entire process is repeated 10 times. By integrating these methodologies, we generate 50 pairs of training and validation sets, enabling a robust evaluation of various interpolation methods. The shuffling method introduces variability and randomness into the selection process, while the 5-fold cross-validation ensures comprehensive coverage of the dataset in a systematic manner.

### 3.3. Machine Learning Algorithms

The primary objective of this study is to identify the most effective machine learning models to generate accurate electric field strength maps in urban environments, thus contributing to advances in environmental monitoring. Using machine learning, our aim is to develop predictive models that not only estimate electromagnetic field (EMF) levels but also incorporate additional urban characteristics, such as population density, urbanization intensity, and specific building features. Machine learning, a subset of artificial intelligence, offers a suite of data-driven methodologies capable of automating model development by training systems to recognize patterns and make predictions with minimal human intervention.

Naturally, the choice to implement machine learning is not coincidental. The enriched dataset requires such methods to uncover hidden correlations. Of course, there are advantages and disadvantages associated with this approach. The disadvantages of machine learning methods lie in the so-called “black-box” issue. Specifically, while these methods can often provide highly accurate predictions, the question of how the model arrived at its conclusions frequently remains unanswered. To address this limitation, we employ the SHAP explanation method. However, the advantages of machine learning methods over simple interpolation techniques are significant. One key benefit is the reduction of errors in estimating the true value, as machine learning methods can facilitate a comparative analysis of hundreds of models. This capability, in turn, allows for greater specialization based on geographical characteristics, something that simple interpolation methods cannot achieve.

In this section, we systematically analyze the machine learning techniques employed, detailing their architectures, hyperparameters, and roles in addressing the complexities of modeling EMF distributions in urban areas.

#### 3.3.1. *k*-NN

The algorithm *k*-nearest neighbors (*k*-NN) is a supervised machine learning method known for its simplicity in regression tasks. The algorithm operates under the assumption that similar data points are likely to exist in close proximity within the feature space. Thus, it identifies the *k* closest data points (neighbors) to a query point in the feature space and makes predictions based on these neighbors. In regression tasks, *k*-NN predicts the output value by computing either the mean or a weighted mean of the target values of neighbors *k*, i.e., $\hat{f}\left(x\right)=\sum_{1}^{k}\frac{f(x_{i})}{k}$.

The performance of *k*-NN depends significantly on the choice of *k*, the distance metric, and the weighting functions for the neighboring data points in the feature space $X^{n}$. In the *k*-NN algorithm, different weights can be assigned to neighbors to emphasize the relative importance of those closer to the query point *x* when making predictions. While uniform weighting ($w_{i}=1\;\forall \;i$) treats all neighbors equally, advanced schemes like inverse distance weighting (IDW) assign weights inversely proportional to distance and so, the $\hat{f}\left(\vec{x}\right)$ at a query location *x* is computed as a weighted average of the known values $f(x_{i})$ at the *n* surrounding neighbors:

(1) $$\hat{f}\left(\vec{x}\right)=\frac{\sum_{i=1}^{n}w_{i}\left(\vec{x}\right)f\left(x_{i}\right)}{\sum_{i=1}^{n}w_{i}\left(\vec{x}\right)}$$

where the weight $w_{i}$ assigned to each known point $x_{i}$ is given by:

(2) $$w_{i}=\frac{1}{d{\left(\vec{x},\;\vec{x_{i}}\right)}^{p}}$$

Alternatively, exponential decay weighting (i.e., $w_{i}=e^{-d_{i}}$), such as Gaussian weighting, assigns weights by the use of a Gaussian function:

(3) $$w_{i}=e^{\left(-\frac{d_{i}^{2}}{2\sigma^{2}}\right)}$$

where σ controls the width of the Gaussian kernel, giving more importance to closer neighbors with weights decreasing exponentially with distance.

Moreover, the *k*-NN algorithm offers the flexibility to alter the distance function, depending on the peculiarities of the dataset and the domain of application. The distance metrics can vary from Euclidean distance:

(4) $$d\left(\vec{x},\;\vec{y}\right)=\sqrt{\sum_{i=1}^{n}{\left(x_{i}-y_{i}\right)}^{2}}$$

which measures the straight-line distance between two points in an (n)-dimensional space to Manhattan Distance $d\left(\vec{x},\;\vec{y}\right)=\sum_{i=1}^{n}\left|x_{i}-y_{i}\right|$, also known as $\left(L_{1}\right)$ norm, or even to more complex distance metrics like Minkowski Distance, widely adaptable for various datasets and domains:

(5) $$d\left(\vec{x},\;\vec{y}\right)={\left(\sum_{i=1}^{n}{\left|x_{i}-y_{i}\right|}^{p}\right)}^{\frac{1}{p}}$$

For the purposes of this study, three hyperparameters were considered. The first is the number of neighbors (*k*), defined as the set {3, 4, 5, 6, 7, 8, 9, 10, 12, 15, 17, 20, 25, 30, 35, 40}. The second is the distance metric, determined by the parameter *p*, with possible values {1, 2, 3}. The third is the weighting scheme, where uniform weights or distance-based weights were applied.

#### 3.3.2. Random Forest

Random Forest is a versatile ensemble learning method widely used in machine learning. It uses a collection of decision trees to improve the accuracy and robustness of predictions for classification and regression tasks, capable of handling diverse datasets with mixed data types and missing values. During training, the method constructs an ensemble of decision trees by training each tree on a random subset of the data obtained through bootstrap aggregation sampling (Bagging) and using a random subset of features for splitting at each node. This dual randomness reduces the correlation among trees, mitigates overfitting, and improves the overall predictive accuracy by ensuring robust generalization results. Random forest achieves enhanced interpretability by evaluating the importance of the feature through the aggregation of impurity reductions in all splits involving a particular feature. Particularly, feature importance is computed based on the reduction in impurity across all trees such as:

(6) $$\mathrm{Importance}\left(j\right)=\sum_{\mathrm{Splits}\;\mathrm{on}\;j}\Delta \mathrm{Impurity}$$

where ΔImpurity is the reduction in impurity due to a split and is computed as:

(7) $$\Delta \mathrm{Impurity}=I_{\mathrm{parent}}-\left(w_{\mathrm{left}}\cdot I_{\mathrm{left}}+w_{\mathrm{right}}\cdot I_{\mathrm{right}}\right)$$

where:

- $I_{\mathrm{parent}}$: Impurity of the parent node.
- $I_{\mathrm{left}}$, $I_{\mathrm{right}}$: Impurities of the left and right child nodes.
- $w_{\mathrm{left}}$, $w_{\mathrm{right}}$: Proportions of samples in the left and right child nodes, respectively.

Thus, the term ΔImpurity measures the “purity gain” obtained by a split, attributing this gain to the feature responsible for the split. Afterwards, the algorithm aggregates the predictions of individual trees, using majority voting for regression $\left(\hat{f}\left(\vec{x}\right)=\frac{1}{N}\sum_{i=1}^{N}{\mathrm{Tree}}_{i}\left(x\right)\right)$, in order to provide a robust final prediction. The hyperparameters in the random forest algorithms dictate the construction and combination of decision trees, ensuring a balance between bias, variance, and computational efficiency. In this study, we have defined the hyperparameter that determines the number of decision trees in each forest ensemble to take values from the list [10, 50, 100]. In addition, the function used to evaluate the split quality employs various criteria. These criteria include squared error, which calculates the mean squared error (equivalent to variance reduction), minimizing L2 loss by using the mean value of each terminal node. The Friedman mean-squared error utilizes the mean squared error with Friedman’s improvement score to identify potential splits. Poisson employs a reduction in Poisson deviance to find optimal splits. In this study, we selected mean squared error (MSE), mean absolute error (MAE), Friedman’s MSE, and Poisson deviation as evaluation metrics.

#### 3.3.3. Neural Networks

Neural networks are computational models inspired by the structure and functionality of biological neurons in the brain, consisting of interconnected artificial neurons organized into layers: an input layer, one or more hidden layers, and an output layer. Each neuron receives multiple inputs, processes them using weights and biases, and applies an activation function to generate an output. Specifically, neural networks’ functionality relies on their capacity to transform inputs into outputs through weighted combinations and activation functions. For example, a neuron’s output is calculated as $\hat{y_{i}}=\sum w_{i}x_{i}+b$, where the weights of the inputs $w_{i}$, $x_{i}$, and a bias *b* determine a weighted sum, followed by an activation function $a=\sigma (\hat{y_{i}})$, such as ReLU, sigmoid, or tanh. Among the different types of neural networks, including recurrent and the well-known convolutional neural networks, feedforward networks (e.g., the multilayer perceptron MLP) are the most commonly and extensively used. Training neural networks involves optimizing their synaptic weights through a process called learning. Depending on the task, learning can be supervised, unsupervised, or reinforced.

One of the most widely used methods for training feed-forward neural networks is the backpropagation algorithm, which iteratively adjusts weights to minimize the loss function, such as mean squared error (MSE) $\mathrm{MSE}=\frac{1}{n}\sum_{i=1}^{n}{\left(y_{i}-{\hat{y}}_{i}\right)}^{2}$ for regression.

Moreover, backpropagation uses gradient descent to compute weight updates layer by layer, ensuring that the network learns to map input patterns to desired outputs. However, training challenges like overfitting and underfitting can arise if the network’s complexity or the training dataset size is not appropriately managed. Generalization, the network’s ability to perform well on unseen data, is critical and often assessed by splitting datasets into training and testing subsets.

Hence, the proper design and training of neural networks are essential for achieving accurate, reliable, and widely applicable solutions. For this study, we tuned the following hyperparameters: the number of hidden layers was set to {2, 5, 10, 20, 50, 100, 200, 300, 500}, and the learning rate was chosen from [0.001, 0.01, 0.1, 0.2, 0.5, 1]. We employed ’adam’ as the solver, which is effective for a broad spectrum of problems, and we experimented with various activation functions, including ’identity’ for linear activation, ’logistic’ for sigmoid activation, ’tanh’ for hyperbolic tangent activation, and ’relu’ for the rectified linear unit.

#### 3.3.4. Decision Trees (DTs)

Decision trees (DTs) are nonparametric supervised learning algorithms employed in both classification and regression tasks. These hierarchical models are represented as tree structures comprising a root node, internal nodes, branches, and leaf nodes. The algorithm begins at the root node, where decisions are made based on feature tests, and continues along branches corresponding to test outcomes until arriving at a leaf node, which provides the final decision or predicted value. Decision trees split data recursively based on feature values, forming branches that lead to distinct decision outcomes.

The structure of a decision tree reflects a flowchart-like design. Each internal node represents a test on a feature or attribute, each branch corresponds to the outcome of that test, and each leaf node represents a class label (for classification tasks) or a predicted value (for regression tasks). To achieve optimal splits, decision trees maximize the homogeneity (purity) of data in child nodes.

For regression tasks, decision trees minimize variance within child nodes to enhance predictive accuracy. The criterion for evaluating splits in regression trees is variance reduction:

(8) $$\mathrm{Variance}\;\mathrm{Reduction}=\mathrm{Var}\left(\mathrm{parent}\right)-\sum_{j}\frac{|D_{j}|}{\left|D\right|}\mathrm{Var}\left(D_{j}\right)$$

where $\mathrm{Var}(D_{j})$ is the variance of the subset $D_{j}$. In the present study, we keep our intervention on DT’s hyperparameters as minimal as possible, and we tune only the parameter that defines the minimum number of samples required to split an internal node, with possible values taking a range of integers from [1, …, 50].

#### 3.3.5. XGBoost (Extreme Gradient Boosting)

Extreme Gradient Boosting (XGBoost) is an advanced machine learning technique that builds upon the gradient boosting framework. It sequentially constructs decision trees, where each tree aims to minimize errors from previous iterations, and it is particularly effective in tasks like classification, regression, and ranking.

Specifically, the method optimizes a regularized objective function:

(9) $$L\left(t\right)=\sum_{i=1}^{n}l\left(y_{i},{\hat{y}}_{i}^{\left(t\right)}\right)+\sum_{t=1}^{T}\Omega \left(f_{t}\right)$$

where $l(y_{i},{\hat{y}}_{i}^{\left(t\right)})$ is the loss function measuring the difference between the true value $y_{i}$ and the prediction ${\hat{y}}_{i}^{\left(t\right)}$, and $\Omega \left(f_{t}\right)=\gamma T+\frac{1}{2}\lambda \sum_{j=1}^{T}w_{j}^{2}$ is the regularization term penalizing the number of leaves *T* and the magnitude of leaf weights $w_{j}$. This regularization reduces overfitting and improves model generalization. Gradients $\left(g_{i}\right)$ and second-order Hessians $\left(h_{i}\right)$ of the loss function are computed to guide optimization such as:

(10) $$g_{i}=\frac{\partial l(y_{i},{\hat{y}}_{i})}{\partial {\hat{y}}_{i}},\;h_{i}=\frac{\partial^{2}l\left(y_{i},{\hat{y}}_{i}\right)}{\partial {\hat{y}}_{i}^{2}}$$

These derivatives are used to update leaf weights and minimize the overall objective.

Moreover, XGBoost achieves high computational efficiency and scalability through parallelized tree construction, sparsity-aware optimization, and out-of-core computation for handling large datasets. It also handles missing data by learning optimal split directions during training. The algorithm offers flexibility through custom loss functions and hyperparameters such as learning rate, maximum tree depth, and regularization terms [30].

Additionally, the interpretability of XGBoost is enhanced by feature importance metrics derived from impurity reduction, such as gain and coverage. However, careful hyperparameter tuning is essential for optimal performance.

For XGBoost, the term lambda is a regularization parameter that controls the L2 regularization term on the leaf weights in the model. It helps reduce model complexity and prevent overfitting by penalizing large leaf weight values. A higher lambda is useful for overfitting models, while a lower lambda can be beneficial for underfitting models. The learning rate (lr) controls the step size during optimization, determining how much the model adjusts the weights with each boosting step. A higher learning rate accelerates training, but can lead to overfitting or instability. In contrast, a lower learning rate improves accuracy, but slows down training. The maximum depth parameter controls the depth of each decision tree. Deeper trees capture more complex patterns but increase the risk of overfitting, while shallower trees are less prone to overfitting but may underfit. For smaller datasets, limiting depth can prevent overfitting, while for larger datasets, increasing depth allows for capturing more complexity. Finally, the number of estimators defines the number of trees in the model. More estimators enable the model to fit more patterns but increase training time and potential overfitting. Each tree sequentially corrects the errors of its predecessors.

In the present study, we set the number of estimators to be from the following list {100, 300, 800, 900}, the learning rate to be from the list {0.08, 0.15, 0.20}, the maximum depth to be from the list {7, 8, 9, 10}, and for the L2 regularization term, which controls overfitting, the options are {1, 2, 5, 10}.

#### 3.3.6. Linear Regression

Linear regression is a methodology where we try to fit a linear model (Equation (11)) called hyperplane to the data by minimizing the residual sum of squares (RSS) between the observed values of the target variable and the values predicted by the model.

(11) $$y=\beta_{0}+\beta_{1}X_{1}+\beta_{2}X_{2}+\cdots +\beta_{p}X_{p}+\epsilon$$

The linear regression model attempts to solve an optimization problem where it tries to find the coefficients $\beta_{0},\;\beta_{1},\;\ldots ,\;\beta_{p}$ that minimize the sum of squared differences between the observed values $y_{i}$ and the predicted values ${\hat{y}}_{i}$, based on the features $X_{1},\;X_{2},\;\ldots ,\;X_{p}$. The optimization problem that the linear regression model solves can be written as:

(12) $$\mathrm{minimize}\;\sum_{i=1}^{n}{\left(y_{i}-{\hat{y}}_{i}\right)}^{2}=\sum_{i=1}^{n}{\left(y_{i}-\left(\beta_{0}+\sum_{j=1}^{p}\beta_{j}X_{ij}\right)\right)}^{2}$$

where $y_{i}$ is the actual value of the target variable for the *i*-th data point; ${\hat{y}}_{i}=\beta_{0}+\beta_{1}X_{i1}+\beta_{2}X_{i2}+\cdots +\beta_{p}X_{ip}$ is the predicted value; *n* is the number of data points.

### 3.4. Accuracy Criteria

Numerous numerical methods exist for evaluating modeling errors, with one of the most widely used being the mean square error (MSE). Ideally, the MSE value should be zero. This metric involves squaring the differences between predicted and actual values before averaging them, ensuring that all errors are positive and assigning greater weight to larger discrepancies. This characteristic often aligns MSE more with user concerns about significant errors. Additionally, we use the root mean square error (RMSE), calculated as the square root of the MSE. RMSE provides an error measure in the same units as the original data, offering a more interpretable representation of the magnitude of the error. In this study, we rely on RMSE for error evaluation.

Root mean squared error is the square root of mean squared error, which provides a measure of the average prediction error in the same units as the data we are trying to predict. The formula is the following:

(13) $$\mathrm{RMSE}=\sqrt{\frac{1}{n}\sum_{k=1}^{n}{\left(y_{i}-{\hat{y}}_{i}\right)}^{2}}$$

RMSE, as an error index, provides an interpretable measure of prediction accuracy, penalizes large errors, and aligns with the objectives of minimizing prediction deviations. These characteristics make it a robust choice for evaluating and comparing our regression models.

### 3.5. Shapley Additive Explanations

The more complex the design of a machine learning model, the harder it is to understand. Deep learning is a prime example, where neural networks involve complex math that can be challenging for non-experts to grasp. As artificial intelligence and machine learning are used more and more in various applications, the need to explain how these models work has become increasingly important. This has led to the development of new techniques such as LIME [31] and SHAP [32], which bring explainability in machine learning to the field. To understand the new framework, let us first clarify what we mean by ’model’ in this framework.

A set X⊂R is called *linearly separable* if there exists a hyperplane *H* such that *H* can separate the set *X* into two subsets *A* and *B*. More formally, for any two points $x_{1},\;x_{2}$ in *X*, if $x_{1}$ belongs to subset *A* and $x_{2}$ belongs to subset *B*, then *H* can be represented as a linear function such that:

(14) $$H\left(x\right)=w^{T}x+b$$

where *w* is called the weight vector. If H(x)>0, then *x* belongs to subset *A*, otherwise, it belongs to subset *B*. Imagine we have data to sort into two categories, like categories A and B. A common approach is to represent these data on a dimensional surface, like a plane. Each data point represents a dot, and its color shows its category.

Now, we can introduce the idea of a model. In this context, a model is like a dividing line on this plane that separates the dots in category A from those in category B. Mathematically, this line can be represented by an equation such as the following:

(15) $$m\left(x\right)=a_{1}x_{1}+a_{2}x_{2}+\ldots +a_{n}x_{n}+c$$

Imagine all the possible dividing lines that we could draw on the plane to separate the data. These lines together form a category that we can call “models”. Our goal is to find the optimal dividing line among them, following certain rules, often minimizing some kind of error.

Now, let us introduce a new idea in machine learning: the “explanation model”. Based on SHAP [32], it is another model itself, but simpler and easier to understand than the original complex one.

We can think of it like this: sometimes it is easier to analyze a problem using a simpler version. Similarly, we can transform the original data *x* (such as lowering the resolution of an image) into a simpler format $x^{\prime }$ using a transformation $x=h_{x}\left(x^{\prime }\right)$, while keeping the important details for the task.

Once we have these simplified data, we can use the explanation model to understand how the original model works. This explanation model is typically simpler, making it easier to understand how the model arrives at its decisions. Our framework uses a specific type of explanation model that relies on adding up the contributions of *additive feature attribution methods,* which have an explanation model that is a linear function:

(16) $$g\left(z^{\prime }\right)=\phi_{0}+\sum_{i=1}^{n}\phi_{i}z_{i}^{\prime }$$

where $z^{\prime }$ are binary variables, *n* is the number of simplified input features, and $\phi_{i}$ is called the attribute effect. In the framework of SHAP, the main aim is to understand the impact of each feature on the model’s output. This involves assessing how the output of the model changes as we vary the input features. The mathematical background of this method has its origin in game theory, more specifically the *Shapley regression values*, which express the importance of each feature for linear models. This method calculates each attribute’s effect as follows:

(17) $$\phi_{i}=\sum_{S\subseteq F\setminus \{i\}}\frac{|S|!\left(|F|-|S|-1\right)!}{|F|!}\left[f\right]$$

where $f_{A}$ is the model over the subset A⊆F of feature set *F*. All these methods, which belong to additive feature attribution methods, have the following properties:

- Local accuracy: When the transformation $h_{x}\left(x^{\prime }\right)$ is identified with *x*, then the explanation model $g(x^{\prime })$ matches with the original model, i.e., $f\left(x\right)=g\left(x^{\prime }\right)=\phi_{0}+\sum_{i=1}^{n}\phi_{i}z_{i}^{\prime }$.
- Missingness: Simply, if $x_{i}^{\prime }=0$, then $\phi_{i}=0$. This means that when $x_{i}^{\prime }=0$, this feature has no attributable impact. Missingness says that a missing feature gets an attribution of zero.
- Consistency: The values remain constant unless there is a change in the contribution of a feature. More importantly, the consistency property says that if a feature becomes more important in making predictions, its Shapley value should also increase or stay the same.

## 4. Results

### 4.1. Model Selection Results

Table 2 presents a detailed comparison of the performance of the five machine learning algorithms in predicting values with optimal hyperparameter configurations. The performance metrics reported in this section are based on the evaluation performed on the test dataset, which constitutes 20% of the total data and is evaluated based on the mean root mean square error (RMSE) measured in volts per meter (V/m). This ensures that the results reflect the ability of the models to generalize to unseen data and are not biased by the training process. Among the models, random forests emerged as the most accurate, achieving the lowest RMSE of 1.26, optimized using the Poisson deviation criterion and 100 decision trees. XGBoost demonstrated competitive accuracy with an RMSE of 1.30, utilizing the regularization term (L2) of 10, 100 estimators, a learning rate of 0.08, and a maximum tree depth of 10. k-nearest neighbors (k-NN) achieved an RMSE of 1.34, using 40 neighbors, Manhattan distance metric (*p* = 1), and a non-uniform weight function, underscoring its ability in non-linear regression tasks. Decision trees recorded an RMSE of 1.47 by optimizing the minimum number of samples required for splitting at 46. Finally, neural networks performed slightly higher error with an RMSE of 1.51, trained using 100 hidden layers, the Adam solver, the tanh activation function, and a learning rate of 1. The results indicate that ensemble methods like random forests and XGBoost outperform individual models, while simpler algorithms such as decision trees and k-NN provide effective but slightly less accurate alternatives. Neural networks, despite their complexity, exhibit potential for further optimization in this task.

### 4.2. SHAP Explanations

**Figure 4:**
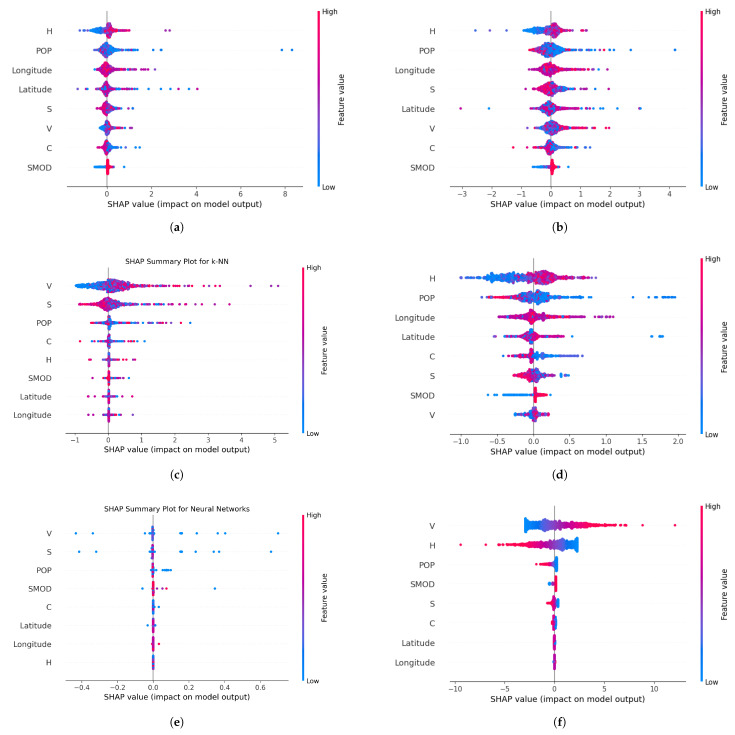
SHAP Summary plot for each machine learning method. (**a**) SHAP summary plot for random forest models. (**b**) SHAP summary plot for XGBoost models. (**c**) SHAP summary plot for k-NN models. (**d**) SHAP summary plot for decision tree models.(**e**) SHAP summary plot for neural network models. (**f**) SHAP summary plot for linear regression models.

The SHAP plots (Figure 4a–f) reveal insightful trends on the importance of features in the prediction of the strength of the electromagnetic field.

Figure 4a,b show the beeswarm plots for the two best-performing models, specifically the random forest and XGBoost models. These plots highlight the importance of variables in predicting electric field strength. The two most significant factors for prediction are building height (H) and population density (POP). Lower building heights (H) are associated with positive SHAP values, as indicated by increasingly red points that extend to the right. In contrast, higher population density values (POP) are associated with negative SHAP values, with the increasingly red points extending to the left. Similarly, the decision trees model (Figure 4d) shows a consistent trend. This indicates that EMF in lower building areas have lower predicted values. The reverse is seen in population density, where lower values lead to higher EMF predictions. In contrast, Figure 4c,e present the beeswarm plots for the k-NN and NN models. These models reveal that the lower surface area values (S) are associated with positive SHAP values, evidenced by the increasingly blue points extending to the right. However, higher surface area values correspond with negative SHAP values, as shown by the increasingly red points extending to the left. For volume (V), the trend is reversed: lower volume values are linked to negative SHAP values, whereas higher volume values are associated with positive SHAP values. This indicates that EMF in areas of lower building volumes have lower predicted values. The reverse is seen in surface areas, where lower values lead to higher EMF predictions. Finally, the linear regression model also highlights volume (V) as the most important factor, showing a similar pattern, where lower volume values are associated with negative SHAP values, and higher volume values correspond to positive SHAP values. However, the second most significant factor, building height (H), differs from the previous models. For linear regression, lower building height values (H) are linked to negative SHAP values, with the increasingly blue points extending to the right.

## 5. Discussion

These EMF maps provide valuable information to policy makers, urban planners, and researchers, empowering data-driven decision making in areas such as public health, infrastructure development, and urban management. For example, the above authorities could be assisted by predictive EFM maps for building construction. Many materials in modern buildings, such as steel or metal, can attenuate the propagation of electromagnetic fields used by mobile telecommunication systems. Although they can also induce higher levels of electromagnetic radiation in the urban environment, they can include various electromagnetic sources such as Wi-Fi routers, smart devices, and other wireless communication technologies [33,34]. Regarding public health, EMF maps can be used as a guide to protect the general population from exposure to EMF, ensuring that EMF strength values comply with the guidelines of the World Health Organization (WHO) and the International Commission on Non-Ionizing Radiation Protection (ICNIRP, 2010).

More specifically, in our research, evaluation of 566 machine learning models was carried out, including random forests, k-nearest neighbor (kNN), neural networks, XGBoost, and decision trees, to predict the strength of the electric field (EMF). The analysis revealed that random forests, XGBoost, and k-NN were the most effective algorithms, particularly when including geographic features as predictive variables. Random forests consistently demonstrated the highest performance, achieving a root mean square error (RMSE) of 1.26. XGBoost followed closely with an RMSE of 1.30, and k-NN achieved an RMSE of 1.34. In contrast, decision trees and neural networks exhibited slightly lower performance, with RMSE values of 1.47 and 1.51, respectively. These findings suggest that ensemble methods, such as random forests and XGBoost, are well suited to predict the strength of EMFs, possibly due to their ability to capture complex relationships within the data. k-NN, while simpler, also proved effective when combined with relevant geographic features.

In conclusion, both random forests and XGBoost offered better performance than the other machine learning models. Although neural networks are powerful models, they are more prone to overfitting when training data are limited, and they may struggle to effectively generalize in high-dimensional spaces. In contrast, random forests and XGBoost, through their ensemble methods and ability to capture complex, non-linear relationships, provide robust performance in our situation. Their relative simplicity and capacity to handle large amounts of data with fewer concerns about overfitting make them strong candidates for electric field strength prediction.

In order to deeper understand the model’s decision-making process, we employed SHAP (shapley additive explanations), a powerful technique for interpreting machine learning models. Our SHAP analysis reveals that the importance ranking of characteristics in tree-based machine learning models differs significantly from those of k-NN, neural networks, and linear regression models. This indicates a multidimensional nature of the problem, necessitating a more granular analysis tailored to the specific factors that influence each method. A promising avenue for future research would be to conduct a comparative analysis in areas characterized by buildings of tall heights or large volumes, assessing model performance, and identifying key contributing variables in such contexts. Our findings demonstrate the significant impact of different machine learning methods on the importance of characteristics and corroboration of our existing scientific understanding. For example, the results confirm the influence of high-rise buildings on electromagnetic field values.

Our research makes a significant contribution to the field of environmental monitoring by developing highly accurate and dynamic maps of the strength of the electric field using advanced machine learning techniques. By combining a comprehensive dataset with explainable AI methods, we have identified key factors affecting the strength of EMF, leading to the development of robust and reliable predictive models. These EMF maps provide valuable information for policymakers, urban planners, and researchers, empowering data-driven decision-making in areas such as public health, infrastructure development, and urban management. The findings of this study demonstrate the transformative potential of machine learning in addressing complex environmental challenges, paving the way for more informed approaches to monitoring and managing electromagnetic exposure in urban environments.

## Figures and Tables

**Table 1:** Comparative analysis of related works.

Research Paper	Machine Learning Tools	Region Area
[15]	CNN	0.065536 km^2^
[17]	U-Net and CGAN	0.0025 km^2^
[18]	LocUNet (CNN)	0.065536 km^2^
[19]	GNN-MDAR	0.000025 km^2^
[20]	NN	0.000144 km^2^
[23]	GNN	1 km^2^
[16]	CNN	0.065536 km^2^
Current research	DT, RF, NN, XGBoost, k-NN, LR	>50 km^2^

**Table 2:** The optimal combination of hyperparameters for each algorithm.

Algorithm	Best Hyperparameters	Mean RMSE V/m
Random Forests	Criterion = Poisson deviance; number of trees = 100	1.26
XGBoost	L2 regularization term = 10; number of estimators = 100; learning rate = 0.08; maximum depth = 10	1.30
k-NN	Number of neighbors = 40; metric = Manhattan (*p* = 1); weight function = not uniform	1.34
Decision Trees	Minimum number of samples = 46	1.47
Neural Networks	Number of hidden layers = 100; solver = adam; activation function = tanh; learning rate = 1	1.51
Linear Regression	-	1.72

## Data Availability

Data are contained within the article.

## Abbreviations

The following abbreviations are used in this manuscript:

RMSE	Root Mean Square Error
DT	Decision Tree
RF	Random Forest
NN	Neural Network
k-NN	k-Nearest Neighbor
XGBoost	Extreme Gradient Boosting
